# Health System Transformation Playbook and Unified Care Model: an integrated design, systems & complexity thinking approach to health system transformation

**DOI:** 10.3389/frhs.2023.1157038

**Published:** 2023-08-02

**Authors:** Ken Wah Teo, Yun Hu, Kwee Tiang Chew, Wee Yang Pek, Hong Choon Chua, David Bruce Matchar, Yeuk Fan Ng

**Affiliations:** ^1^ Corporate Development, Khoo Teck Puat Hospital, Yishun Health, National Healthcare Group, Singapore, Singapore; ^2^ Hospital Administration, Khoo Teck Puat Hospital, Yishun Health, National Healthcare Group, Singapore, Singapore; ^3^ CEO’s Office, Khoo Teck Puat Hospital, Yishun Health, National Healthcare Group, Singapore, Singapore; ^4^ Programme in Health Services and Systems Research, Duke-NUS Medical School, Singapore, Singapore; ^5^ Saw Swee Hock School of Public Health, National University of Singapore, Singapore, Singapore

**Keywords:** health system transformation, design thinking, systems thinking, complexity thinking, complex adaptive systems, care model, change management, healthcare organization

## Abstract

Health system transformation is a complex journey that often results in unintended consequences. Existing methods to drive health system transformation have intrinsic limitations which impede successful implementation in local contexts. The Health System Transformation Playbook is a design-, systems-, and complexity-thinking enabled methodology to systematically design, prioritize and test health system and services transformation actions, anchored on iterative story telling, model building and pathfinding processes that tackles the scale of socially and technologically complex adaptive systems through time. The Unified Care Model and its associated cascade of models are examples of ongoing application of Health System Transformation Playbook in a regional population health system in Singapore. Use of Health System Transformation Playbook enables stewards of health systems to gain a more systematic and coherent understanding of health systems and services planning and organization development, to accelerate transformation towards people-centered, integrated and value-driven health systems.

## 1. Introduction

Health systems across the world are facing challenges related to sustainability and resilience stemming from a confluence of demand- and supply-sided factors including ageing populations and shortages of healthcare infrastructure and workforce (1). Large-scale health system transformation, through a series of interventions aimed at coordinated, system-wide change across organizations and providers, is needed (2). However, research on such transformation is limited, and instead, innumerable small-scale change initiatives carried out by single healthcare organizations or business units have been implemented and documented in published literature (2). These efforts often end in failure to aggregate or scale into system-wide transformation (3).

Herein, we propose the Health System Transformation Playbook (HSTP) as methodological guidance for large-scale health system transformation, based on an integrated design thinking, systems thinking and complexity thinking approach. We further describe a case study of HSTP application in the development and implementation of the Unified Care Model (UCM) and its associated cascade of models to accelerate transformation towards a people-centered, integrated and value-driven regional population health system in Singapore.

## 2. Theoretical methods

### 2.1. Health systems as complex adaptive systems

The health system is an open system composed of multiple and powerful independent agents and organizations whose behaviors are often guided by personal interests and institutional factors (4). Such conflicts can results in heterogeneity of health systems development and performance outcomes. Agents and organizations are constantly adapting and learning, with the overall system self-organizing, and systemic patterns and outcomes are emergent, rather than being the result of rational design and systems optimization (5). The relationships between systemic structures in a health system are numerous, non-linear and interdependent, and feedback is dynamic and delayed. Without any single point of control, plus judgements about cause-and-effect are less certain before the fact, this makes coherent health systems development and transformation difficult (6). Additionally, a number of subsystems within health systems have inherently high thresholds for change but are extremely influential due to their numerous casual links vis-à-vis the rest of the health system. As a result, the health system exhibits path dependence and lock in as critical parameters and tipping points to produce transformative change is seldom reached.

For transformation to succeed, efforts must recognize that health systems are complex adaptive systems and address the attendant problems which arise in such systems. First, a human-centered problem solving approach is needed as health systems are fundamentally serving the needs of end-users and are socially constructed systems (7). The views of all stakeholders should be considered, and solutions should meet the needs of end-users. It is also important to bring these stakeholders along in the transformation journey to increase agency for change, and this means training and gathering stakeholders as co-designers. Second, the scale of the health system must be considered (8). Improvements need to optimize the whole system rather than just its parts. This requires consideration of the hierarchies of systems, including the interrelationships between systemic structures in health systems. Finally, the problem-solving approach in health system transformation must consider uncertainty over time and complexity (9). Innovations to transform health systems need to remain coherent and synergistic despite their introduction at different time points and into different sub-systems continuously. Incremental-only innovations that result in premature convergence and lock-in into paths that are less transformative or with potential negative effects should be minimized.

Indeed, the issue of many interventions across multiple settings failing to translate into meaningful whole health system improvement has been hypothesized to be because they were developed in or for a specific context, and cannot simply be generalized to local operational and organizational contexts (10). We posit that these contextual factors are reflections of the features of complex adaptive health systems. A review by Kaplan et al., identified 66 distinct contextual factors associated with successful implementation of conceptual models across settings (11) and Rogers et al., proposed a categorization of contextual factors across levels of systems (12). To tailor implementation strategies to specific contexts, Powell et al., suggests to integrate theory, evidence, and stakeholder perspectives while recognizing the challenges of doing so in complex adaptive systems (13). Intervention mapping (14), concept mapping (15), conjoint analysis,(16) and system dynamics modeling (17) have also been proposed as more rigorous methods to derive tailored implementation strategies (18, 19).

### 2.2. Analysis and synthesis of primary theories

Drawing on our regional population health system transformation experience, as well as understanding of work by authors in the field of complex adaptive systems and implementation science, we hypothesize that an integrated design thinking, systems thinking, and complexity thinking approach that iteratively adapts interventions to context is required to accelerate large-scale health system transformation. Sturmberg et al., have echoed the call for this integrated approach, although methodological guidance remains elusive (20).

Health systems are human-designed, human-centered, and founded on the collective mental models of its members (21). However, in large-scale health system transformation, the views of important stakeholders including patients, providers and policy-makers are often times insufficiently considered (22). This can result in interventions failing to meet the needs or solve the problems of the end user i.e., patients or providers. It may also contribute to a decades-long gap between development and implementation (23). Design thinking is a systematic process of innovation through empathetic engagement of stakeholders most knowledgeable about the current-state, collaboration with stakeholders to brainstorm and critically appraise solutions, iterative prototyping, and rapid scale-up of optimized solutions (24). Despite its potential, conventional design thinking has been applied to mostly single disease, process and stakeholder perspectives only on a small scale (25). In large-scale change, design thinking has instead played a limited role (26, 27). A review of design thinking approaches being applied to health care interventions by Altman et al., also demonstrated mixed success and a clear evidence gap, with 19 of 24 studies being observational, 14 having a small sample size, only two being good quality (25).

The health system is also comprised of a hierarchy of systemic structures (28) and organizational structures (29). Important health system performance patterns such as experience, outcomes and value emerge from the interactions between these structures (21). Systems thinking is a philosophy and discipline of thinking about structural and interdependent causes of conditions and consequence of actions that helps to address problems at scale (30). It differs from reductionist approaches by viewing systems as wholes, where changes to parts of systems may not yield anticipated changes in wholes (31). A large variety of systems thinking tools have been developed to build models hypothesizing systemic and dynamic causes of problems and to implement and evaluate change scientifically (32). Additionally, the World Health Organization proposes a health system model comprising six interdependent sub-systems (33), and guidance to harness systems thinking to understand health systems, anticipate emergent behaviors, and contextualize interventions that accelerate systems strengthening (8). A review by Jha et al. assessed 26 studies of large-scale health system transformation that were guided by systems thinking and identified two critical issues (32). First, only nine studies examined all six World Health Organization sub-systems. Second, no studies critically analyzed the interrelationships between all sub-systems sufficiently to inform whole system dynamics, citing limited capacity to account for a large number of stakeholders and variety of contexts.

Indeed, health systems contain many components which are highly interrelated, resulting in a dynamic and ever-changing system (34). Complexity thinking is a school of thought within systems thinking developed to address the known unknowns, unknown unknowns, and the uncertainty arising from these features. In contrast to general systems thinking, complexity thinking aims to sense make within an environment characterized by low certainty and low agreement relationships between actors about cause-and-effect (35). Snowden’s Cynefin sensemaking framework is one example which proposes to probe-sense-respond with safe-to-fail experiments to enhance insights in a complex environment before more definitive action (36, 37). Plsek et al. further suggests the following principles for working with complexity: minimum specification, and generative relationships with the positive use of attractors and a constructive approach to enable change. These minimum specifications, termed Simple Rules by Best et al., are summarized as necessary rules for large-scale health system transformation, including engaging individuals at all levels in leading change efforts, establishing feedback loops, attending to history, engaging physicians, and involving patients and families (2). Similarly, Polarity Management by Johnson has been proposed as a tool to navigate tension within systems and enable more generative relationships and constructive approaches (38, 39). Finally, Khan et al., and Lanham et al. proposed to go beyond recognizing contextual factors to instead focus on optimizing the interdependencies between them (40, 41). However, agents, structures and change ideas constantly enter and leave the system, resulting in emergence more often than planned design, and both intended and unintended effects may become locked-in into the system (42). We posit that applying complexity management alone potentially risks lock-in to unintended paths with high costs of reversing negative effects. Without systemic design, the myriad of health system transformation actions may also fail to converge coherently into a better health system.

**Figure 1:**
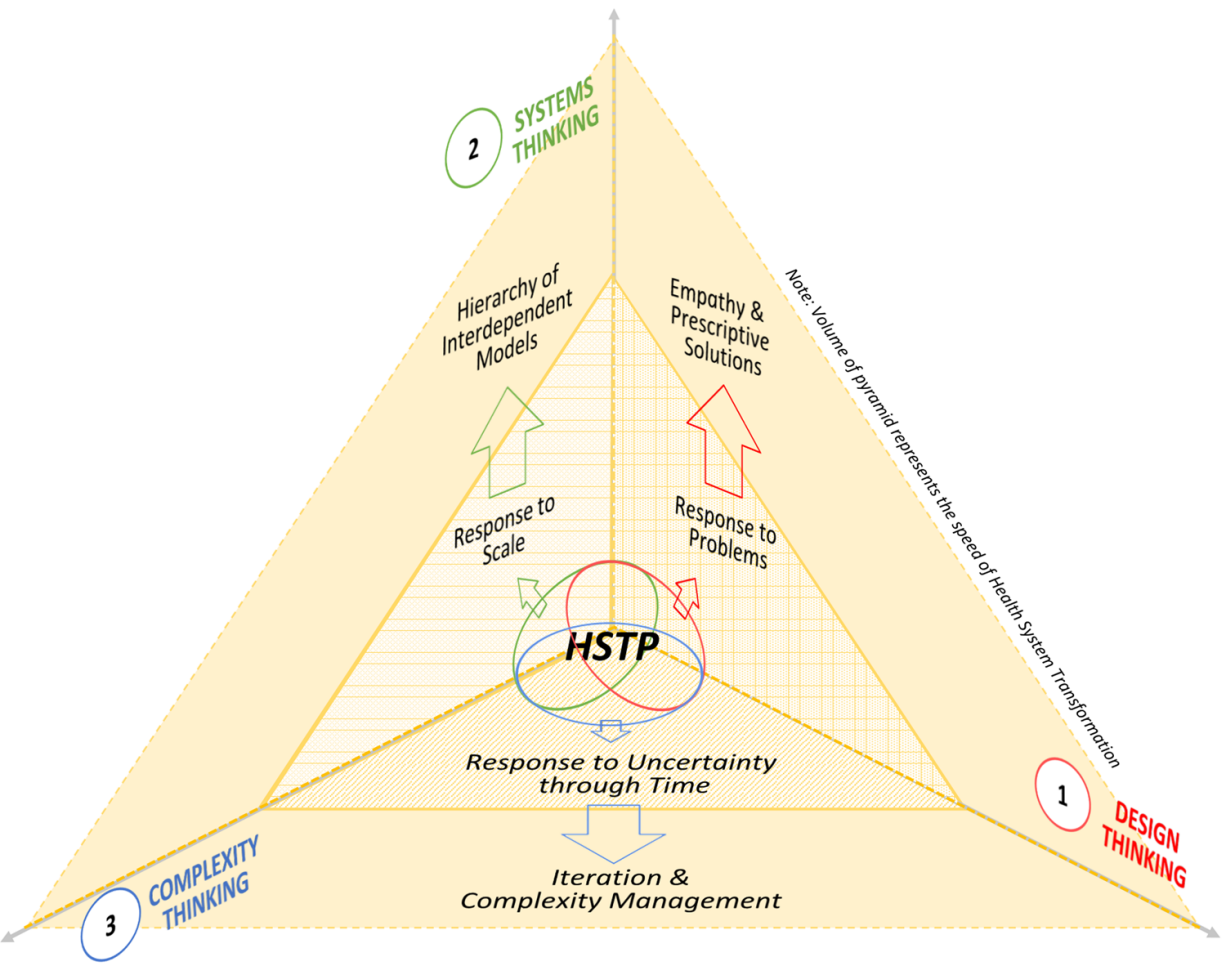
Health System Transformation Playbook—A design, systems, complexity thinking-enabled approach to accelerating health system and services transformation.

In this regard, design thinking, systems thinking, and complexity thinking each offer established approaches to health system transformation with distinct strengths. However, we recognized the methodological and implementational limitations inherent to these single methods. We found it counter-productive to use each approach alone or deploy their stepwise activities sequentially in health system transformation as this accrues the benefits of just one approach and limitations would not be addressed. Herein, we propose an integrated design thinking, systems thinking, and complexity thinking methodology for health system transformation (Figure 1).

Design thinking applies inductive, deductive, and abductive reasoning to analyze, prescribe and iterate prototype solutions (43). It solves problems of end-users based on desirable future-state models (44). The empathetic, strengths-based and bottom-up approach involving multiple stakeholder perspectives strengthens agency (45). However, being limited to the current problem and subject to biases inherent in stakeholders gathered, unintended consequences may result when other agents, components and interdependencies within whole systems are not consciously considered (46). Conventional design thinking alone is therefore insufficient in addressing the scale of health system transformation. Systems thinking provides a solution for this as it replaces reductionism with expansionism and analysis with synthesis, to identify systemic structures and interdependencies in whole systems (47). This generates broader descriptive models of current-states and potentially recommends more holistic prescriptions. However, such approaches may be seen as top-down and de-emphasize the agency needed for innovation and change (48).

Integrating design thinking and systems thinking, designer-facilitators may create more interactive and generative planning environments for stakeholder-designers. By honoring differing perspectives within larger system contexts, stakeholder ownership is strengthened and a shared vision of system-wide improvements is developed (45, 46). Indeed, the emerging field of systemic design supports integration of resources across levels of systems and between ecosystems by bringing together multidisciplinary stakeholders to design more systemically and thus co-create a future-state that is better able to meet end-user needs (49). In this approach, the facilitator is a systemic-designer drawing upon methods and tools drawn from both disciplines (50).

However, it is not possible to bring the whole health system into a room and work through the expanse of stakeholder data and experiences. It is also difficult to appreciate continuously the adapting holism and interdependencies of an entire system through time, as demanded by an integrated design thinking—systems thinking approach. Complexity thinking stresses that new ideas are understood only through retrospective coherence (51). Therein lies the complementary strength of complexity thinking to probe the ever-changing realities of stakeholders and thereby identify adjacent possible solutions and preserve agency (52).

We propose therefore that an integrated design thinking, systems thinking, and complexity thinking approach has better potential to prioritize actions with greater degrees of certainty and agreement but prevent premature convergence by holding open the paths of change towards more holistic interventions as prescribed by models obtained through systemic design. This combined approach is encapsulated in the HSTP, which offers novel methodological guidance for large-scale health system transformation through time. Herein, we also illustrate the Yishun Health UCM and its associated hierarchy of future-state models as a case study of the HSTP process and result.

## 3. Results

### 3.1. Health System Transformation Playbook

HSTP is an integrated design thinking, systems thinking and complexity thinking enabled three-step planning and organization development methodology for health system transformation. HSTP prescribes an overarching process bringing together numerous other tools to enable and accelerate health system transformation (Table 1, Appendix 1).

**Table 1:** Health System Transformation Playbook checklist.

Step One: Story Telling—Seeing the System
• Gather stakeholders • Encourage sharing of personal, team or leadership stories such as strengths, challenges, data, trend, innovation or ideas • Distil the change ideas in these stories • Document a list of existing systems potentially impacted by the change ideas and organize them into a hierarchy • Document a list of existing models of the same systems.
Step Two: Model Building—Understanding the System
• Clarify the potential impact to systems and the causal pathways between them • Clarify the potential impact to existing models and improvement plans • Name any new systems that need to be created and add this to the list of future-state systems • Distil a list of design principles for each future-state system • Articulate a model or refine the existing future-state model for each future-state system
Step Three: Pathfinding—Working with the System
• Clarify where the current system is versus future models • Prescribe interventions to deploy the models (bring current state stakeholders and systems into the future) • Evaluate all model prescribed interventions against organizational and operational context to determine level of certainty and agreement • Generate list of prioritized actions in four Action Classes and track implementation • Repeat Step One when prioritized actions generated new stories or data

Step One is “Story Telling—Seeing the System”, which gathers stakeholders interested in improving their health system. Stakeholders share personal, team or leadership stories about strengths, challenges, data, trends, innovations, or ideas, and a facilitator helps to distill change ideas from these stories. The group then proceeds to clarify the existing systems and models impacted, the purposes, boundaries, contents, and leads of these systems and models, as well as the hierarchy in relation to the larger health system. Useful tools for this step include ethnographic methods (53), Appreciative Inquiry (54), and vertical and horizontal facilitation approaches (55) to generate rich pictures (56) or giga/systemic maps and visualizations (57) of current-state systems. This enables stakeholders to broaden their individual views and create a shared view of their current-state systems, including their operative mental models and future-state models of the same systems.

In Step Two, “Model Building—Understanding the System”, this shared view is deepened through facilitated conversations and group model building. The potential impact of change ideas on the shared understanding of future-state models, i.e., the purpose, boundary and contents, is assessed. Potential impacts on any ongoing tailored interventions in existing systems that were previously prioritized to evolve future-state systems are also reviewed. Facilitation should also create shared understanding of the potential impact of change ideas on interactions between future-state models, as well as impact on the interdependencies between previously prioritized interventions. Positive and negative effects that may arise are thereby distilled. Effects are akin to design principles, with positive and negative effects being desirable and undesirable features respectively, that future systems must manifest or avoid. Based on these design principles, stakeholders initiate model building to co-create or refine a cascade of future-state models hypothesized to improve health system performance. may also name new systems and create corresponding models. The purpose of future-state models is to help stakeholders define a shared vision of the future-state system as it is impacted by the change ideas, and so doing prepare stakeholders for Step Three, “Path Finding—Working with the System”.

Accordingly, future-state models can take different forms depending on the system involved and its hierarchy within the health system. For example, higher in the hierarchy of systems, models tend to be conceptual or structural and less detailed. Lower in the hierarchy, models increasingly delineate operational processes and are more detailed. Useful tools for this step can therefore range from concept maps (58), causal loop diagrams (59), and systemic design templates (60) for future-state systems, to logic models (61), empathy maps (62), and value stream maps (63) of the future-state processes.

In model building, it is important that stakeholders distinguish current- and future-state models. Stakeholders should always start iterating from and on existing future-state models to incorporate the selected change ideas generatively so the shared understanding that stakeholders have of their future health system is increased. Additionally, the impact of change ideas on future-state systems and models should be evaluated starting at the top of the hierarchy, indicative usually of where the future primary desired outcome will be accrued. Working down the hierarchy, stakeholders should reach an understanding of the systems and models impacted, and of those constraining the intended future outcome. New design principles should be introduced only if the performance of higher systems will concurrently be improved. Measurements should be adopted to track the hypothesized improvement of future-state systems where practicable.

In Step Three: “Path-finding—Working with the System”, stakeholders clarify where current-state systems are in relation to future-state models, and brainstorm interventions to drive health system transformation. Stakeholders then assess model prescribed interventions against organizational and operational contexts to ascertain the level of agreement and certainty and generate a list of prioritized actions in four Action Classes (Table 2). Useful tools to analyze model prescribed interventions in relation to the local context and improve the process for classifying prioritized actions into Action Classes include aforementioned tools for tailoring implementation strategies such as menu-based choice methods, discrete choice experiments and conjoint analysis techniques (18). Here, any ongoing actions such as tailored interventions in existing systems that were previously prioritized by now outdated models must be revisited to determine alignment with the model prescribed interventions prescribed by the cascade of future-state models.

**Table 2.** Health System Transformation Playbook Action Classes.

**III. Higher Agreement-Lower Certainty** • Deeper research to assemble evidence & evaluative data • Introduce limited development pilots	**I. Higher Agreement-Higher Certainty** • Mobilize resources • Prioritize as work plan items/ projects
**IV. Lower Agreement-Lower Certainty** • Deeper research to assemble evidence & evaluative data • Introduce safe-to-fail probes • Deprioritize temporarily	**II. Lower Agreement-Higher Certainty** • Engagement & conversations • Collaborative projects • Relationship building

Interventions prescribed by the cascade of future-state models in the four Action Classes through this process can form the organization’s health system transformation plan. Where there is higher agreement and certainty, resources should be mobilized, constraints alleviated, and Class I prioritized actions implemented. Prescribed interventions with lower agreement or certainty should not be abandoned, nor should the future-state models be redesigned at this stage. Instead, actions that raise agreement or certainty can be prioritized. Exaptive innovation through collaborative projects are examples of Class II prioritized actions that draws upon stakeholder differences to generate breakthrough change aligned with prescribed interventions while building relationships and expanding agreement. Class III prioritized actions includes deeper research to assemble evidence, or limited development pilots to test specific hypotheses that can raise certainty. Finally, Class IV safe-to-fail probes can test reality and gauge acceptability amongst different stakeholders and identify groups with higher agreement. With new stories emerging from driving a health system transformation plan with prioritized actions in four different Action Classes, stakeholders then repeat HSTP steps.

### 3.2. Case study of iterative Yishun Health Unified Care Model development through the Health System Transformation Playbook

#### 3.2.1. Singapore and Yishun Zone context

Singapore’s health system has consistently been rated highly worldwide (64) due to impressive outcomes achieved at relatively low total health expenditure (65). However, an ageing population with increasing burden of disease, medical advances, and rising workforce costs (66) have prompted questions about sustainability. Between 2012 and 2017, healthcare expenditure rose exponentially from $13 billion to $22 billion (65). Recognizing this, the Singapore government articulated three fundamental policy shifts, the “Three Beyonds”: beyond healthcare to health, beyond hospital to community, and beyond quality to value (66). This emphasized preventive care, appropriate siting of care in the community than hospitals, and improving quality of care sustainably.

In 2017, the National Healthcare Group radically reorganized into the three integrated care organizations of Yishun Health, Central Health and Woodlands Health, with the renewed purpose of caring for a community’s health than for patients’ healthcare needs alone. Khoo Teck Puat Hospital, a 795-bed acute hospital, Yishun Community Hospital, a 428-bed sub-acute, rehabilitative, and palliative care hospital, and Admiralty Medical Centre, an ambulatory care center, were reorganized into Yishun Health. As an integrated care organization caring for 320,000 residents within the regional population health system of Yishun Zone in Northern Singapore, Yishun Health’s role expanded beyond healthcare service provision, to caring collaboratively with care partners within the zone. This provided Yishun Health impetus for accelerating health system transformation into a people-centered, integrated and value-driven regional population health system.

Operating within this context, Yishun Health initiated numerous health system and services transformation initiatives to improve residents’ health and wellbeing, integrate care and optimize outcomes and value of the Yishun Zone Regional Population Health System. In our transformation journey, the Yishun Health Chief Executive Officer, supported by planning, development, and engagement teams from corporate, hospital and community departments organized numerous thematic workshops and meetings. In the process, residents, care partners, and staff of institutions within Yishun Zone embarked on facilitated story telling conversations, group model building exercises, and prescribed and analyzed interventions to prioritize actions.

#### 3.2.2. Step one: story telling—seeing the Yishun Zone system

Using HSTP Step One methods and tools, our story telling initiatives were aimed at ensuring ongoing engagement of a wide range of stakeholders across our health system through time. To date, more than three large health system retreats involving more than 100 heads of departments and several hundred staff have been conducted. Smaller and regular resident and patient focus group discussions, care partner engagement meetings, transformation platform meetings and workshops by different staff groups continue to be ongoing. A Yishun Zone Population Health Survey with qualitative and quantitative components to discover residents’ needs is currently in progress. Stories from these continuous engagements sessions of stakeholders throughout our system have revealed the need for better integrated care plan development for residents and patients, need for strengthened ownership of these populations with enhanced communication and accountability for outcomes, need for hassle-free access to value-driven care, and need to ensure continuous improvement across the whole system.

Facilitation vertically across teams and longitudinally through time then helped to continuously deepen, clarify, and build a shared understanding of current-state systems. For example, our stakeholders have collectively defined the existing systems and associated models within Yishun Health and arranged these in a hierarchy of macrosystem, mesosystems, and microsystems (67) impacting health system outcomes. Our macrosystem was defined as the Yishun Zone Regional Population Health System, which overlaps and interacts with other macrosystems such as the local community and social systems. Our health system also operates within the larger National Healthcare Group and Singapore health system and is subject to their policies. Internally, we recognized the service delivery system and five other mesosystems, corresponding to the World Health Organization’s six sub-systems. The existing institution-specific service delivery systems of Khoo Teck Puat Hospital, Yishun Community Hospital, and Admiralty Medical Centre were led by the Chief Medical Officer. Their purpose was to meet their patients’ healthcare needs, measured using conventional disease- and hospital-based metrics such as disease-specific mortality rates and length of inpatient stay. These systems boundaries corresponded largely to physical boundaries of each institution, and the system contents included patients, care teams and institutional infrastructures. Although service delivery systems are perpetual without definable start or end, they were typically experienced by patients as short care episodes corresponding to durations of clinic attendance or inpatient stay. Accordingly, the microsystems within each service delivery system were largely site-based and specialty-specific, such as the Khoo Teck Puat Hospital inpatient care system or the orthopedic clinic system. These current-state features manifest in turn, in the prevalent mental models and ongoing improvement plans, which is to deliver care after disease has arisen, meet disease-specific healthcare needs, mostly within institutional physical boundaries.

#### 3.2.3. Step two: model building—understanding the Yishun Zone system

The ongoing body of HSTP Step One activities has to-date generated more than 1,000 change ideas related to improving resident and patient care needs and their experience of health and healthcare in Yishun Zone. These change ideas were analyzed thematically to aid stakeholders in their articulation of design principles and development of a cascade of future-state health system models through iterative group model building activities.

For example, we recognized that the change idea “Three Beyonds” fundamentally impacts the design that our service delivery systems must take going forwards. We diagnosed the practices, structures and mental models that were no longer adequate to achieve our revised system purpose. We then distilled design principles to integrate our future-state service delivery system. These are the positive effects we must incorporate, including existing features that continue to have utility (Table 3), and the unintended negative effects we must guard against in response to the change ideas (Table 4). For example, traditional service provision adopted a medicalized approach for patients that began largely after disease has set in. This will be insufficient going forward. While medicalized care within institutional settings remains relevant, biopsychosocial (68), and needs- (69) and assets-based (70) approaches to well-being and health that includes prevention, promotion and public health throughout the life-course will be necessary for the entire Yishun Zone population. However, implementing this design principle require attention to mitigate potential negative effects such as loss of personal responsibility for one’s own well-being and health, over-medicalization of self, caregiver and community-based care, and healthcare professionals experiencing loss of agency and becoming disengaged.

**Table 3:** Design principles for Yishun Zone Regional Population Health System's future-state integrated service delivery system.

S/N	Existing Practices, Structures and Mental Models	Design Principles for Future-State Integrated Service Delivery System
1	Medicalized approach that addresses clinical issues when disease or crisis sets in, for patients who seek treatment at healthcare institutions	Biopsychosocial (68), needs-(69) and assets-based (70) approach to well-being and health throughout a persons’ life-course, for the entire Yishun Zone population
2	Services provision primarily by healthcare professionals using a clinical and service quality approach	Residents, caregivers and communities drive self-care, and also participate in care planning and co-creation of care delivery with providers that employ strength-based (71) and relationship-based approaches
3	Individual provider needs assessments and care plans formulation	Single integrated needs assessment and care plan to meet residents’ needs
4	Interactions among providers and healthcare institutions are case by case, professional and operational	Interactions among providers and care institutions are based on population needs, and are organizational and strategic
5	Care episode defined from time-of-arrival to time-of-departure from care systems defined by physical boundaries of institutions	Care episode defined from time-of-identification to time-of-resolution of needs, and system boundaries defined by types of needs
6	Coordination achieves efficiency and effectiveness of services delivery within institutions	Coordination achieves efficiency and effectiveness of care systems in discovering and generating health assets while meeting care needs, across all sites and transitions of care
7	Providers and institutions optimize institutional outcomes and their own cost-per-unit of services delivery	Carers and institutions form an integrated care organization and jointly optimize resident-centered outcomes and value for populations

**Table 4:** Potential unintended effects of Yishun Zone Regional Population Health System's future-state integrated service delivery system transformation.

Unintended Negative Effects to Guard Against
1. Loss of personal responsibility for one’s own well-being and health 2. Over-medicalization of self, caregiver and community-based care 3. Reduced quality of care and patient safety for acute and complex needs 4. Loss of privacy as care is shared across a network of providers 5. Excessively complex care plans that are not implementable 6. Uncertainty about who is responsible for the care plan 7. Healthcare professionals experiencing loss of agency, becoming disengaged 8. Loss of flexibility in choosing care or service providers 9. Excessive paperwork and general bureaucracy 10. Micro-management of providers outside institutions with loss of institutional identity 11. Cost-cutting exercises masquerading as value-driven care 12. Poorly articulated, implemented, and communicated value-based care assessments

**Figure 2:**
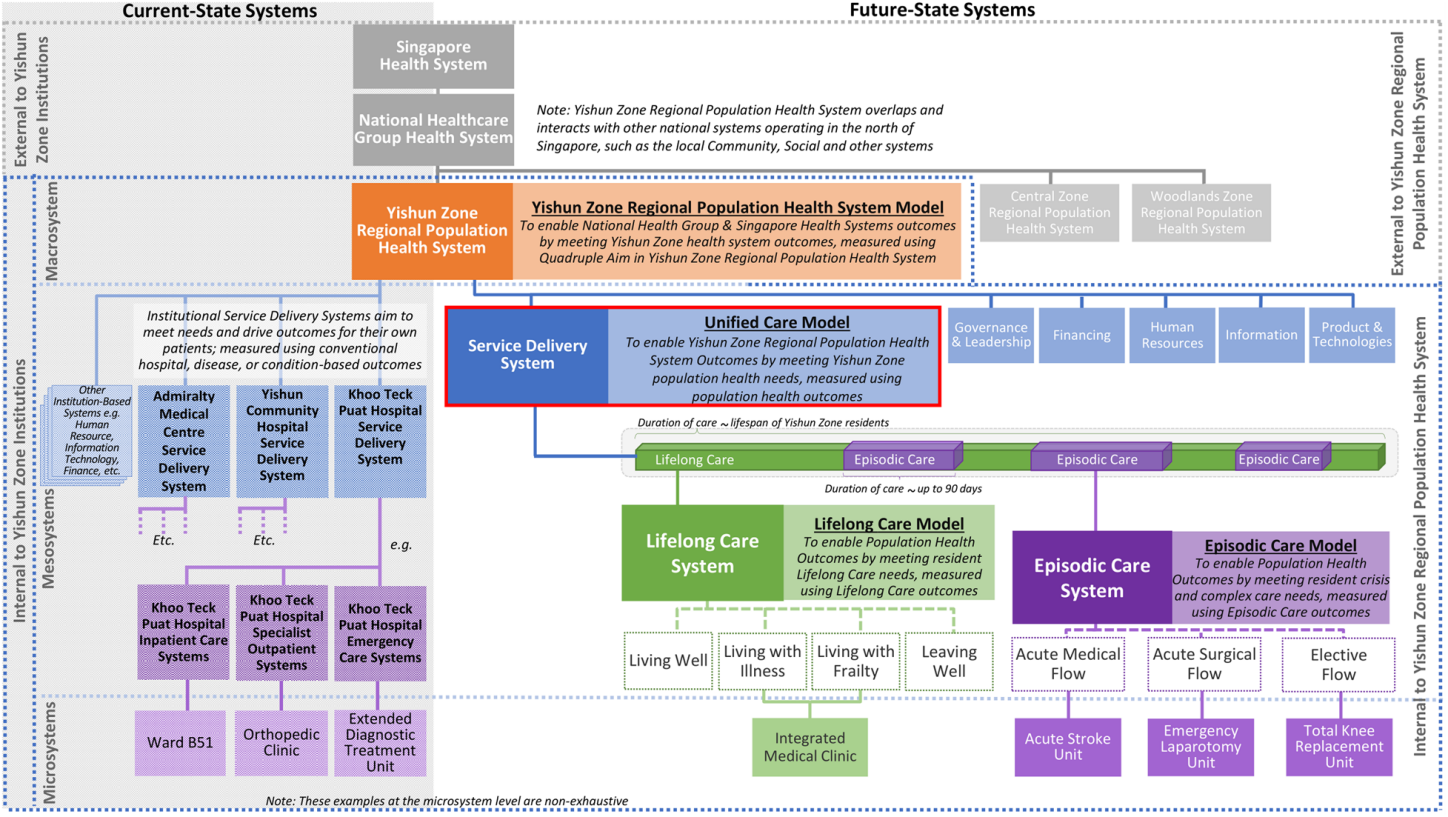
Yishun Zone Regional Population Health System's hierarchy of systems and models.

The UCM and its cascade of future-state models (Figure 2) is the result of our group model building exercise led by Yishun Health Chief Medical Officer. The UCM is a model of care that ensures that all residents have a One Care Plan emphasizing a fit and healthy life, and hassle-free access to dignified, safe, and value-driven care (72) by collaborative teams and networks (73). The UCM represents Yishun Health’s aspiration to co-create with all staff and our communities, the highest form of integrated care (74) that is person- and community-centered (75), and built upon collective strengths and shared goals, trust, and relationships.

The UCM embraces the expanded purpose of meeting population health needs, measured through metrics such as resident health-related experience, quality-of-life, and levels of protective health factors. Patient care outcomes of provider organizations continue to be important but are subsidiary within these broader outcomes. The costs of attaining outcomes will be measured across all providers to optimize resident and patient value. The spatial boundary of the UCM is wider and mirrors Yishun Zone Regional Population Health System. Responsibility for outcomes will thus be shared between all UCM contents, including residents, families, communities, care partners, such as primary care clinics and social welfare organizations, and Yishun Health. Temporally, the UCM will be experienced by all residents throughout their life-course.

Within the UCM are subsidiary integrated care models of Lifelong Care and Episodic Care systems. Lifelong Care seeks to meet the totality of health needs of the Yishun Zone population and is defined temporally from birth to death, and spatially, so long as the person is a resident within Yishun Zone. Accordingly, Lifelong Care is where Yishun Health will work with residents to discover their biopsychosocial needs and assets for well-being and health, to jointly develop and execute their One Care Plans. Lifelong Care focuses on enabling capabilities for self and family care within caring communities, while working with care partners to guard against over-medicalization of care and resident loss of autonomy and privacy. Within the Lifelong Care system are microsystems representing smaller, mutually exclusive sub-populations with distinct needs such as those living with chronic illness, living with frailty, or at their end-of-life. Each resident will be enrolled into one Lifelong Care microsystem only, and in totality these sub-populations are therefore exhaustive of the Yishun Zone population. Our Integrated Medical Clinic is an example of a future-state service model for such microsystems (76).

Episodic Care is a sub-system within Lifelong Care and its purpose is to meet the sporadic health needs arising from acute crisis and/or complex elective medical events throughout a resident’s life-course. Episodic Care is defined temporally from start to resolution of such needs. Episodic Care is where Yishun Health will work to guard against cost-cutting at the expense of quality, and mitigate potential loss of agency and engagement amongst existing healthcare professionals. Episodic Care is sub-segmented into Acute Medical, Acute Surgical, and Elective flow systems, within which are microsystems caring for smaller populations with distinct Episodic Care needs such as Acute Stroke, Emergency Laparotomy and Total Knee Replacement. Being a sub-system of Lifelong Care, all Episodic Care plans necessarily occur within the context of the residents’ ongoing Lifelong Care One Care Plans. This elaborate division of Lifelong Care and Episodic Care systems into microsystems defined around populations of person-centered needs will ensure that care teams work across institutional boundaries unambiguously towards integrated and value-driven outcomes.

Our model building exercise also accorded the UCM recognition as the highest model in our hierarchy of health system subsystem models, since broadly speaking, a health system functions to care for people, so the pinnicle system in a health system should be its care or service delivery system. Accordingly, efforts are underway to articulate design principles for our other five future-state subsystems to be aligned with UCM design principles. For example, our Governance & Leadership mesosystem must go beyond clinical governance of disease- and institution-based patient segments, to include health system governance of needs-based population segments across all provider organizations. Likewise, our Health Information mesosystem must go beyond hospital-based systems operating institutional data models, to population-based systems operating an overarching Unified Data Model that is person-, population- and health system-centered.

Iterative group model building exercises continue in Yishun Health. Ongoing current efforts are increasingly focused on the development of operational and process models lower in the hierarchy of systems. This will eventually guide care and service delivery transformation based on UCM design principles, enabling residents and patient to experience the UCM directly and attain better health outcomes.

#### 3.2.4. Step three: path finding—working with the Yishun Zone system

Finally, the UCM and its cascade of future-state models, throughout its development and continual iteration, was used to prescribed interventions for Yishun Zone health system transformation. The analysis, discussion, and prioritization of actions based on UCM-model prescribed interventions took place in various large health system retreats and workshops. These prioritization discussions continue regularly in smaller and more regular C-suite conversations, Episodic Care and Lifelong Care Transformation platform meetings, and care partner engagement meetings. Classification of prioritized actions into Action Classes is done by the health systems and services planning and development team and approved by heads of departments and senior management. Relevant analytical tools are in the process of being evaluated for adoption to aid prioritization and classification into Action Classes more systematically.

**Figure 3:**
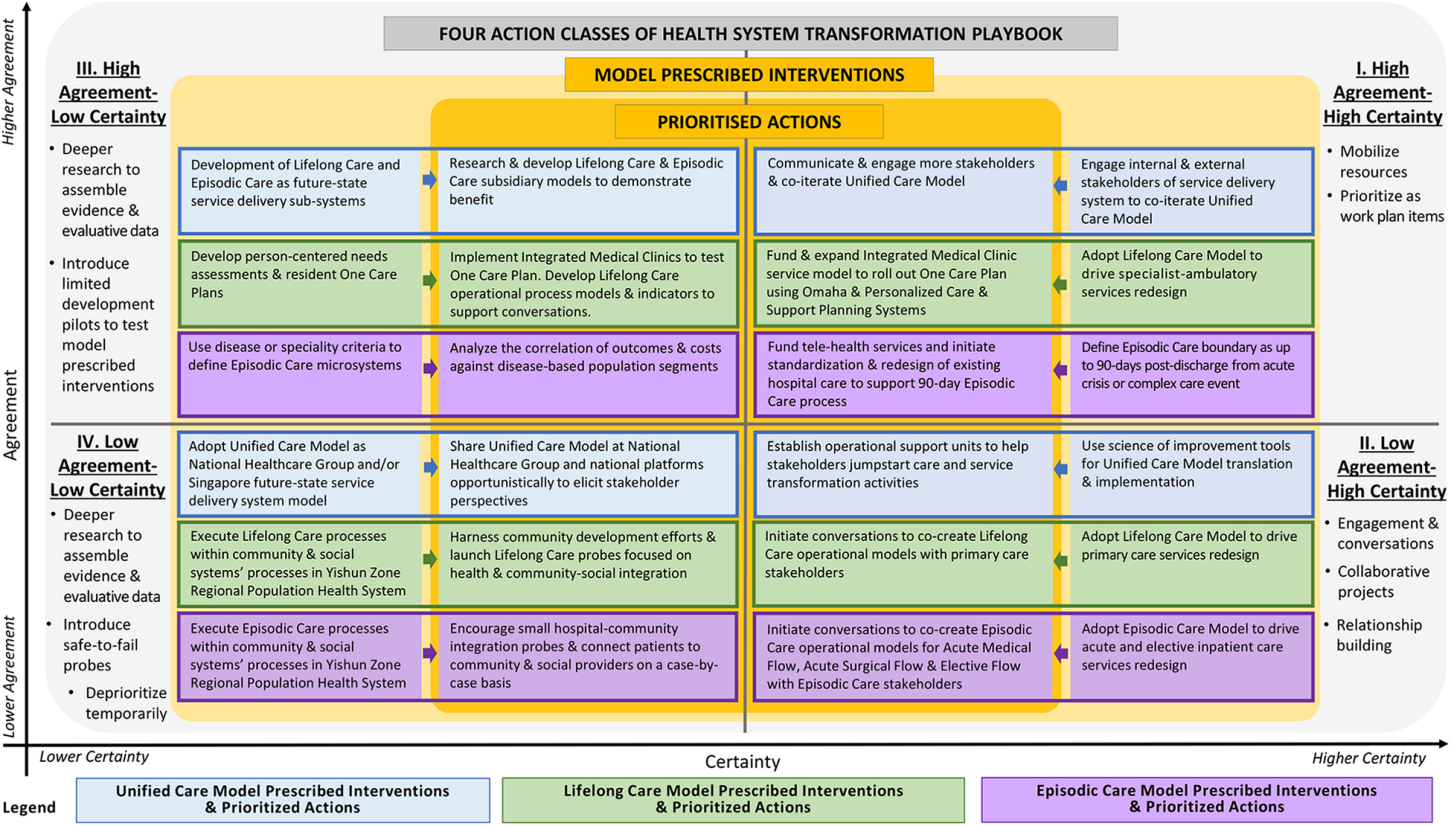
Unified Care Model prescribed interventions, with prioritized actions in Health System Transformation Playbook four Action Classes.

A portfolio of prioritized actions according to local levels of agreement and certainty is also maintained (Figure 3). For example, under Action Class I, the UCM prescribed continuing stakeholder engagements to iterate the UCM itself. We therefore executed briefings and workshops targeting care teams, heads of departments, and care partners to generate greater understanding of UCM and develop model building capabilities. Under Action Class III, the UCM prescribed development of Lifelong Care and Episodic Care as sub-systems of our future-state integrated service delivery system. To raise certainty, we initiated projects to aggregate data from different care teams to exemplify conceptual Lifelong Care and Episodic Care outcomes. We also conducted research into care strategies of Lifelong Care and Episodic Care to develop better integrated services models to guide care and services transformation.

## 4. Discussion

Health systems are large socially constructed complex adaptive systems. Health system transformation can be guided by various principles, frameworks and models as the specific intent, scale, progress and stage of transformation is different in each system. Every health system differs also in stakeholder attitudes and capabilities for transformation. Where there is need and ambition for large-scale or acceleration of whole health system transformation, current approaches are limited (2). First, singular methods and tools employed to enable more limited problem solving or systems change may not work at the scale of the whole health system or through time, will have intrinsic limitations, and do not provide clear enough methodological guidance (77). Second, the principles, frameworks, and models used to guide health system transformation in one context very often cannot be generalized to other operational and organizational contexts. Likewise, the tailored interventions that arise in one context will still fail to translate into meaningful whole system improvement as they insufficiently address causal mechanisms needed for a different context (18). Last, the implementation of interventions is often not explicitly based on the set of approaches appropriate for whole health system change, or implementation had considered only a subset of all the needed approaches (78). There is therefore a need for enhanced implementation science methods that specifies better the mechanisms of change to design and tailor implementation strategies for whole health systems transformation (19), particularly through better integration of theory and evidence, eliciting stakeholder perspectives and participation, and offering clear methodological guidance that can enable replication of approaches across contexts (18). In this regard, we build on Sturmberg’s early guidance on the need to synthesize design thinking, systems thinking and complexity thinking to accrue the benefits of each method while addressing their limitations (18).

First, the HSTP codifies design thinking, systems thinking, and complexity thinking into a simple checklist of stepwise actions anchored on iterative story telling, model building and path finding which brings together a suite of available implementation science tools and methods. The HSTP further provides specific guidance on the types of prioritized actions using four Action Classes, with the process of prioritization enabling systematic deployment of rigorous methods to derive tailored implementation strategies including those described by Powell et al (18). The HSTP therefore provides clear methodological guidance and prescription for health system transformation that more effectively integrates theory and evidence surrounding implementation science methods.

Second, through the HSTP process governed by systemic-designer facilitators, stakeholders are brought together to define the current-state systems and models, clarify the impact of change ideas on these models and systems, align on future-state systems and models, and agree on model prescribed interventions. The HSTP also proposes that model-building always begins with the latest future-state models to ensure new change ideas build upon current health system transformation progress. This secures stakeholder perspectives and participation both throughout the system and through time. Finally, the iterative use of HSTP enables the engagement of numerous stakeholders as stakeholder-designers through time. This fosters organization learning through shared understanding of local systems. It also enhances health system transformation capability in the organization as stakeholder competencies to be systemic-designers and experts in health system implementation science are honed through iterative model building and path finding. It is only through such deeper levels of learning that creates increasing stakeholder awareness of the larger whole, both as it is and as it is evolving, that leads to actions that increasing serve the emerging whole (79). Finally, the specific rules of engagement within the HSTP systematizes more effectively a process of transformation suited for whole health systems, and thereby enables HSTP application across various contexts. For example, the cascade of future-state models that arises through HSTP is a stock of desirable change ideas for the health system that also represents the collective strategic goals, agency, and leadership interest to drive health system transformation. Through the HSTP, higher certainty but lower agreement interventions prescribed by future-state models will not be abandoned and can instead be operationalized when and if the right opportunities arise to lock the system into a more desirable development path, such as after prioritized actions have raised levels of agreement. HSTP can therefore maintain the creative tension between polarities (80) of transformational and sustaining innovations (81) and better guard against incremental-only innovations. We posit that the HSTP therefore enables better designed and tailored interventions for large-scale health system transformation.

By exemplifying the HSTP approach in the development and implementation of the UCM in a regional population health system in Northern Singapore, we further add to the body of implementation science evidence available. Indeed, we demonstrated an enhanced method for designing and tailoring implementation strategies, as well as tracking and reporting of their progress through time (19). Anecdotally, we experienced the benefits of the HSTP process during UCM development and deployment. For example, as we engaged stakeholders from over a hundred departments in various care integration and service planning conversations, the amount of coherence has increased among stakeholders of Lifelong Care and Episodic Care. In effect, the Lifelong Care and Episodic Care models represented enabling constraints for innovation that increased clarity about how different care teams can work together to integrate care and drive services transformation. We also observed gradual emergence of more individuals working coherently as systemic designers for health system transformation beyond the service delivery system, for example, in corporate planning, analytics, and informatics, where UCM was used to redesign corporate work plans, performance management processes, and information technology systems. Finally, the Integrated Medical Clinic that was designed to meet Lifelong Care needs of Yishun Zone residents with complex chronic medical and social challenges represents a prioritized action aimed at raising the level of certainty to enable implementation of the UCM-prescribed Lifelong Care system and One Care Plan at scale.

## 5. Limitations

First, we acknowledge that HSTP is itself a methodology bringing together many other methods and tools, all requiring substantial practice for skillful usage during health system transformation. Additionally, HSTP may require considerable time and effort to implement, and organizational or external pressures may compel health system stewards to adopt ill-conceived but popular quick fixes.

We propose that deep competence in HSTP may paradoxically be simpler to attain than for the entire range of tools and methods potentially required in different situations. Real quality improvement is not possible without profound knowledge (82). Health system stewards must ultimately attain competence in understanding their own systems, understanding the positive and negative effects of change ideas on systems, grappling with their own realities, and charting their own paths forward. HSTP enables this, and provides helpful clarity about the roles of external designer-facilitators versus internal stakeholders as systemic designers that are also experts in implementation science. For example, while external designer-facilitators can be engaged for short durations when specialized expertise is needed, we recommend they operate within a process managed by health system stewards responsible for health system transformation using HSTP.

Second, the effectiveness of HSTP remains contingent on human and system factors. For example, evaluations of positive and negative effects of change ideas, and assessments of levels of agreement and certainty of prescribed interventions, are ultimately judgements by health system stewards. Similarly, stakeholders’ capabilities in applying abductive logic to develop coherent interventions and actions, the quality of stakeholder relationships and their willingness to collaborate during model building are innate system characteristics. As are the competence of existing health system stewards to govern and address secondary effects of system change, such as when prescribed interventions create “winners” and “losers” across sub-systems. Further research into aptitudes and skills that complement HSTP implementation is needed; but all things equal, we posit that iterative use of HSTP will result in the development of systems characteristics that accelerate large-scale health system transformation successfully.

Last, development and implementation of HSTP and our UCM are ongoing efforts. While anecdotal feedback suggests that UCM is helpful for care integration and service delivery system transformation and our health system transformation journey is accelerating, this paper represents our learning-to-date and wider resident and stakeholder engagement, such as with National Healthcare Group, Ministry of Health, and our local community and social partners, is needed. We have also initiated further research to systematically evaluate HSTP and UCM as methodology and model for integrating care and accelerating health system transformation.

## 6. Conclusion

Health system transformation is a complex and long-drawn journey with potentially unintended and costly consequences. HSTP is an integrated design thinking, systems thinking and complexity thinking enabled methodology to systemically design, prioritize and test health system and services transformation actions. HSTP is anchored on an iterative story telling, model building and pathfinding approach that tackles the scale of a socially constructed complex adaptive systems through time. Our experience suggests that HSTP enables health system stewards to accelerate health system transformation while retaining what is valued and without incurring serious adverse secondary effects.

UCM and its associated cascade of models are herein described as an example of the ongoing application of the HSTP for care integration in a regional population health system. Through this paper, we hope health system stewards may engage in health system transformation as more than rhetoric, or a mix of single method approaches, but rather as an integrated learning and development process that engages people in all organizational sub-systems and the broader environment. So doing, stewards may gain a more systemic and practice-based understanding, to catalyze and accelerate health system transformation and organization development towards people-centered, integrated and value-driven population health systems.

## Data Availability

The original contributions presented in the study are included in the article/Supplementary Material, further inquiries can be directed to the corresponding author.
