# Supplementary material for: Health System Transformation Playbook and Unified Care Model: an integrated design, systems & complexity thinking approach to health system transformation
Source: Front Health Serv. 2023 Aug 2;3:1157038. doi: 10.3389/frhs.2023.1157038 (PMC10433688; doi:10.3389/frhs.2023.1157038)
Supplement: Supplementary file 1 [file Table1.docx]

Supplementary Material

**Appendix 1: Health System Transformation Playbook Checklist, Tools & Outputs**

| **Health System Transformation Playbook Check List, Tools & Outputs** | | |
| --- | --- | --- |
| **Steps** | **Relevant Tools**  **(Non-Exhaustive)** | **Definitions of HSTP Outputs** |
| **Step One: Story Telling – Seeing the System** | | |
| □ Gather stakeholders | Appreciative inquiry, world café, focus groups, systems maps, and process maps | 1. **Systems:** A hierarchy of current-state systems impacted by the change ideas, organized such that the system where the primary desired outcome is accrued is positioned at the top. 2. **Leads:** A list of the leaders in charge of the corresponding systems. 3. **Purpose of Systems:** Shared understanding of the purpose of these systems. 4. **Boundary of Systems:** Shared understanding of the physical and/or chronological boundary of these systems. 5. **Contents:** Shared understanding of the constituents of these systems from stakeholders’ perspectives such as people and infrastructure. 6. **Models:** A list of existing models of these same systems. This can be a mental model of the current state, or an agreed future-state model adopted by the team to drive improvement. |
| □ Encourage sharing of personal, team or leadership stories such as strengths, challenges, data, trend, innovation or ideas |  |  |
| □ Distill the change ideas in these stories |  |  |
| □ Document a list of existing systems potentially impacted by the change ideas and organize them into a hierarchy |  |  |
| □ Document a list of existing models of the same systems. |  |  |
| **Step Two: Model Building – Understanding the System** | | |
| □ Clarify the potential impact to systems and the causal pathways between them | Driver diagrams, logic models, value stream maps, and empathy maps | 1. **Impact on Systems & Causal Pathways:** Shared understanding of the potential impact the change idea can have on the list of systems, and on the known interdependencies, interactions, or feedback between these systems. 2. **Impact on Models & Improvement Plans:** Shared understanding of the potential impact the change idea can have on existing current-state mental models, future-state models already adopted by the team to drive improvement, or any agreed improvement activities or ongoing work plans that are based on existing mental models. 3. **Design Principles:** A list of positive and negative effects that future systems must manifest or avoid respectively. 4. **Positive Effects:** A list of intended consequences or desirable features that future systems must aim to have. 5. **Negative Effects:** A list of unintended consequences or undesirable features that future systems must aim to be free from. 6. **Future-State Systems:** Shared understanding of the list of future-state systems, organized into a hierarchy of future systems, along with details of the proposed lead, purpose, boundary, contents, and models. 7. **Future-State Models:** A cascade of future state models of the corresponding future-state systems. Each model is the result of iterative model building, where positive effects are designed into the model and negative effects are designed away. |
| □ Clarify the potential impact to existing models and improvement plans |  |  |
| □ Name any new systems that need to be created and add this to the list of future-state systems |  |  |
| □ Distill a list of design principles for each future-state system |  |  |
| □ Articulate a model or refine the existing future-state model for each future-state system |  |  |
| **Step Three: Pathfinding – Working with the System** | | |
| □ Clarify where the current system is versus future models | Business process re-engineering, service and experience design, and project management tools | 1. **Prescribed Interventions:** A list of desirable, long- to medium-term HST interventions that is prescribed by future-state models. It is generated via stakeholder discussion of the path needed to get from current-state systems to the future-state models of the same systems. It can be reported in the form of an organizational strategic plan for the next 5-10 years. 2. **Prioritized Actions:** A list of short-term HST actions that are generated by considering the levels of agreement and certainty for each prescribed interventions within the organizational and operational contexts. Prioritized actions can be in four Action Classes depending on the level of certainty and agreement. It can be reported in the form of a work plan for the next 1-2 years.   **Four Action Classes of HSTP**   \| **III. Higher Agreement-Lower Certainty**   - Deeper research to assemble evidence & evaluative data - Introduce limited development pilots \| **I. Higher Agreement-Higher Certainty**   - Mobilize resources - Prioritize as work plan items/ projects \| \| --- \| --- \| \| **IV. Lower Agreement-Lower Certainty**   - Deeper research to assemble evidence & evaluative data - Introduce safe-to-fail probes - Deprioritize temporarily \| **II. Lower Agreement-Higher Certainty**   - Engagement & conversations - Collaborative projects - Relationship building \| \|  \|  \| |
| □ Prescribe interventions to deploy the models (bring current state stakeholders and systems into the future) |  |  |
| □ Evaluate all model prescribed interventions against organizational and operational context to determine level of certainty and agreement |  |  |
| □ Generate list of prioritized actions and track implementation |  |  |
| □ Repeat Step One when prioritized actions generated new stories and data |  |  |
